# Primary care clinician perspectives on automated nephrology e-consults for diabetic kidney disease: a pre-implementation qualitative study

**DOI:** 10.1186/s12875-024-02454-w

**Published:** 2024-06-04

**Authors:** Chi D. Chu, Daniel Dohan, Michelle M. Estrella, Michael G. Shlipak, Delphine S. Tuot

**Affiliations:** 1 Department of Medicine, University of California, San Francisco, San Francisco, CA 94110 USA; 2 Department of Medicine, Division of Nephrology, Priscilla Chan and Mark Zuckerberg San Francisco General Hospital, 1001 Potrero Ave, Bldg 100, Rm 342, San Francisco, CA 94110 USA; 3 Kidney Health Research Collaborative, Department of Medicine, University of California, San Francisco VA Health Care System, San Francisco, CA USA; 4 Philip R. Lee Institute for Health Policy Studies, University of California, San Francisco, San Francisco, CA USA

**Keywords:** Diabetic kidney disease, Chronic kidney disease, Nephrology referral, E-consults, Electronic consultation

## Abstract

**Background:**

Many patients with diabetic kidney disease (DKD) do not receive evidence-based, guideline-recommended treatment shown to reduce DKD progression and complications. Proactive electronic consultations (e-consults) are an emerging intervention strategy that could potentially allow nephrologists to provide timely and evidence-based guidance to primary care providers (PCPs) engaged in early DKD care.

**Methods:**

The objective of this study was to explore perspectives about potential barriers and facilitators associated with a proactive e-consult program to improve DKD care delivery. We conducted semi-structured qualitative interviews with PCPs across three different health systems. Interview transcripts were reviewed in a rapid qualitative analysis approach to iteratively identify, refine, and achieve consensus on a final list of themes and subthemes.

**Results:**

A total of 18 interviews were conducted. PCPs across all sites identified similar challenges to delivering guideline-recommended DKD care. PCPs were supportive of the proactive e-consult concept. Three major themes emerged surrounding (1) perceived potential benefits of proactive e-consults, including educational value and improved specialist access; (2) concerns about the proactive nature of e-consults, including the potential to increase PCP workload and the possibility that e-consults could be seen as documenting substandard care; and (3) leveraging of care teams to facilitate recommended DKD care, such as engaging clinic-based pharmacists to implement specialist recommendations from e-consults.

**Conclusion:**

In this pre-implementation qualitative study, PCPs noted potential benefits and identified concerns and implementation barriers for proactive e-consults for DKD care. Strategies that emerged for promoting successful implementation included involving clinic support staff to enact e-consult recommendations and framing e-consults as a system improvement effort to avoid judgmental associations.

**Supplementary Information:**

The online version contains supplementary material available at 10.1186/s12875-024-02454-w.

## Background

Diabetic kidney disease (DKD) is the leading cause of chronic kidney disease (CKD) and kidney failure in the US and is associated with significant cardiovascular morbidity and mortality [1, 2]. Despite the enormous health burden of DKD, many patients with DKD currently do not receive evidence-based, guideline-recommended treatment crucial for reducing DKD progression and complications. In studies examining diverse health systems and population settings, the proportion of persons receiving guideline-recommended medications to slow DKD progression, such as angiotensin converting enzyme inhibitors (ACEi) and angiotensin II receptor blockers (ARB), has consistently been 50–60% without improvement over time [3–6]. Projected increases in diabetes and kidney failure incidence highlight the critical need for innovative population health approaches for improving delivery of optimal DKD care [7, 8].

Previously studied interventions to improve DKD or CKD care have shown mixed success and have included educational programs directed at PCPs, audit-based performance feedback, and electronic health record-embedded clinical decision support [9–15]. A meta-analysis of randomized controlled trials evaluating interventions to improve CKD management in the primary care setting found no benefit to either computer-assisted or education-related interventions, compared to usual care, for the outcomes of improving guideline-concordant ACEi or ARB prescription, proteinuria assessment, or blood pressure control [16]. Implementations of clinical decision support have been hindered by alert fatigue and poor individualization of actionable recommendations to PCPs, leading to inconsistent improvements in care [13–15].

Proactive electronic consultations (e-consults) are an emerging system-level intervention strategy that could potentially allow nephrologists to provide timely and evidence-based guidance to PCPs engaged in early DKD care. In contrast to the traditional referral or e-consult framework that requires PCPs to initiate the consultation, proactive e-consults involve a strategy to identify patients who could benefit from specialist input at the system-level, after which specialists would conduct a targeted chart review and provide their recommendations to PCPs as an e-consult [17]. Strategies to identify patients for DKD management may include laboratory criteria (e.g., elevated albuminuria) or validated kidney failure risk prediction models [18], which can be applied at the health system level to identify the target population for proactive e-consults. Patients identified in this manner can then be individually reviewed by nephrologists, resulting in individualized recommendations that are delivered to the patient’s PCP in the form of an e-consult message. The proactive nature, which does not require PCPs to initiate the e-consult request, is the key distinguishing feature compared with traditional e-consults or referral mechanisms. E-consult documentation would leverage existing documentation infrastructure in the electronic health record. E-consult contents, including the specialist recommendations as well as subsequent communications between the PCP and the specialist, become part of the permanent electronic health record. E-consults are visible to all clinicians accessing the chart, and in many health systems, visible to patients as well.

Potential advantages of this e-consult strategy include (1) the proactive nature, which does not rely on PCPs’ explicit recognition, diagnosis of DKD, or decision to refer, (2) expert specialist input, which allows recommendations to be more tailored to individual patients compared with what is possible with clinical decision support rules, and (3) the interactive capability, which allows PCPs to electronically discuss management with specialists to clarify or further tailor treatment recommendations to specific patient scenarios. Conversely, potential disadvantages of a proactive e-consult strategy include unclear acceptability of the proactive approach among PCPs or patients and logistics of primary care workflows for implementing unsolicited e-consult recommendations, which may depend substantially on how primary care clinics are set up and operate within different health systems.

Versions of proactive e-consults have been implemented in a few settings, such as for osteoporosis management in a regional veteran population and for high-risk CKD care in the Kaiser Permanente Hawaii health system [19, 20]. These proactive e-consult programs demonstrated only modest effectiveness in improving treatment rates. The objective of this study was to explore perspectives from PCPs practicing in three different health systems about potential barriers and facilitators associated with proactive e-consults. These findings may provide valuable pre-implementation insights to inform the optimal design and development of a proactive e-consult program to improve guideline-concordant DKD care delivery.

## Methods

### Study design and population

We conducted semi-structured qualitative interviews with PCPs. Participants were purposively sampled across practice sites in three different health systems, each of which comprised multiple primary clinic sites: an academic health system, an urban public safety net health system serving under-insured and uninsured populations, and a Veterans Affairs (VA) health system. Each health system also had its own network of specialists, including nephrology. We approached medical directors at primary care clinic sites in each health system to identify potentially eligible PCPs, who were then invited to participate via email. In some clinics, PCPs were invited by email from the study team after being referred by their director; in other clinics, the director shared the study invitation broadly to PCPs. Eligible PCPs were defined as clinicians actively practicing primary care. We used broad inclusion criteria to mimic clinicians in a busy primary care practice. Physicians (MD or DO), nurse practitioners, and physician assistants were eligible, and we did not require a minimum percent professional effort dedicated to primary care as long as it was not zero. Due to limited independent practice experience, trainees were not included. Verbal consent was obtained before each interview. The study protocol was approved by the University of California, San Francisco Institutional Review Board (#22-37188).

### Data collection

Semi-structured interviews were conducted with Zoom video conference by a physician-investigator (C.D.C.) trained in interviewing methodology. All interviews were completed between February 2023 and October 2023. Interviews lasted up to one hour and were audio recorded and transcribed. An interview guide was used to facilitate discussion about potential implementation of a proactive nephrology e-consult program for DKD management (Item S1). Questions were designed to elicit challenges in delivery of guideline-recommended DKD care, potential barriers and facilitators to a proactive e-consult intervention, and suggestions for optimizing the intervention’s effectiveness and integration into PCPs’ existing workflows. Demographic and practice-related information were self-reported by participants, including: gender, race and ethnicity, years in practice, training background, and percent time dedicated to direct patient care. Sample size was guided by interim assessment of interview data for thematic saturation, and in similar prior work, we reached thematic saturation at 14 interviews [21].

### Analysis

Data were analyzed using a rapid qualitative analysis methodology [22, 23]. Interview transcripts for each participant were reviewed and consolidated into a matrix organized by broad themes and subthemes as they emerged from the data related to PCPs’ responses to proactive e-consults. For this process, three members of the research team (C.D.C., D.D., D.S.T.) held regular in-person meetings to iteratively review transcripts and identify, refine, and achieve consensus on a final list of themes and subthemes. Representative quotations were extracted to illustrate subthemes. We followed the Consolidated Criteria for Reporting Qualitative Research checklist for reporting of qualitative research [24].

## Results

A total of 18 interviews were conducted among 6 academic, 8 safety net, and 4 VA PCPs (Table 1). The median number of years in practice was 6 (interquartile range 4–12). Training backgrounds included internal medicine (*n* = 13), family medicine (*n* = 4), and nurse practitioner (*n* = 1).

**Table 1:** Participant characteristics (*n* = 18)

Characteristic	
Female gender, n (%)	15 (83)
Race, n (%)	
Asian	6 (33)
Black	1 (6)
White	10 (56)
Multi-racial	1 (6)
Health system, n (%)	
Academic	6 (33)
Safety net	8 (44)
Veterans Affairs	4 (22)
Years in practice, median [IQR]	6 (4, 12)
Training background, n (%)	
Family Medicine	4 (22)
Internal Medicine	13 (72)
Nurse Practitioner	1 (6)
Percent time in direct patient care, n (%)	
≥75%	6 (33)
50 − 74%	6 (33)
<50%	6 (33)

To provide contextual background, we will first summarize the barriers to delivery of guideline-recommended DKD care as reported by PCPs in their current practice before presenting the results of thematic analysis. PCPs identified a number of barriers including (1) difficulty staying up to date with clinical practice guidelines, (2) challenges related to new medications and medication management, and (3) limitations due to poor patient access and continuity of care (Table 2).

**Table 2:** Illustrative quotations for challenges providing guideline-recommended diabetic kidney disease care

Site type	Quotation	Quotation no.
Safety net	I’ll also say that just even since I’ve completed residency 10 years ago, there’s just been a flood of new classes of medications coming out for diabetes, right? And just the lack of conferences and grand rounds…Just stuff like that when you fall out of the academic milieu you’re just on your own a lot more, so that can just be uncomfortable…even if you know what the new guidelines are, which probably a lot of people don’t. (PCP1)	1
Academic	There are no set of professional guidelines that I personally follow. I have some old notes from residency that I use in terms of like reminding me what labs to monitor and with what frequency. But I don’t think I’ve ever actually referred to professional guidelines or professional society guidelines for CKD, if I’m being completely honest. (PCP13)	2
VA	I came out of training when [SGLT2 inhibitors] became, right as it was becoming super popular…And so I didn’t quite understand fully, and I’m still trying to struggle to understand the mechanism of renal protection…I do know some of the adverse effects to look out for. But I would say for myself, I’m not feeling super confident on what patients should I definitively be looking at for SGLT2 inhibitors, at least outside the realm of diabetic management. (PCP15)	3
Safety net	My experience has been it’s really hard to drive pushing [guideline-indicated] medicines as hard as we need to push them, partly because the patient population we take care of takes a long time to develop trust with us. And the last thing we want to do is, when they get in, say…we’re gonna give you…an SGLT2, and we’re gonna…drive your lisinopril up as high as we can get it, even though…your blood pressure looks okay…it’s hard for patients to take multiple, multiple changes from us. (PCP4)	4
Safety net	We have limited resources to see patients and then, there are often barriers to access…So we see them and then we may not see them again for another year or two because they are marginally housed, or experiencing homelessness, or they fall off and then they get other insurance and they come back, and we have this revolving door. So I think that creates a bit of a problem trying to follow any guidelines. (PCP4)	5
VA	Getting [guideline-based treatment recommendations] on a patient that I’ve seen like once and haven’t been able to really get a hold of…it doesn’t quite always help. Especially if there’s like other things that are active, like my patients with chronic open wounds and active substance use disorders. You know, it’s like prioritizing specific things like that. (PCP15)	6

*Abbreviations* SGLT2 = sodium-glucose cotransporter 2; VA = Veterans Affairs

These challenges were consistently expressed by PCPs across all three health systems. One frequent challenge was keeping up with the volume of evolving clinical practice guidelines across multiple chronic conditions, especially after their training period (Quotation [Q]1; Table 2). Many reported having internalized elements of DKD care during their training (e.g., knowing to use ACEi or ARB) but reported low explicit awareness of specific guideline criteria, organizations creating clinical practice guidelines, and updates to those guidelines. (Q2; Table 2). Limited comfort with prescribing and patient counseling for new drug classes, such as sodium-glucose cotransporter 2 (SGLT2) inhibitors, was also cited as a barrier to guideline-recommended care (Q3; Table 2). PCPs reported that prescribing indicated medications could be deferred if patients were having difficulty adhering to their current regimen. This was compounded by reported difficulty counseling patients on why changing their regimen or adding new medications was indicated, particularly for patients whose blood pressure and diabetes were well-controlled on existing regimens:

My experience has been it’s really hard to drive pushing [guideline-indicated] medicines as hard as we need to push them, partly because the patient population we take care of takes a long time to develop trust with us. And the last thing we want to do is, when they get in, say…we’re gonna give you…an SGLT2, and we’re gonna…drive your lisinopril up as high as we can get it, even though…your blood pressure looks okay…it’s hard for patients to take multiple, multiple changes from us. (Q4; Table 2)

Limitations in patient access to care and continuity of care were also consistently reported as significant barriers impeding safe prescribing and monitoring practices needed for optimal DKD care; even when patients are able to attend appointments, more active medical and/or social issues may be prioritized over optimizing chronic disease management (Q5 & Q6; Table 2).

With regard to the topic of proactive e-consults, PCPs were generally supportive of the concept as a mechanism for facilitating guideline-recommended care delivery and ensuring optimal treatment for patients with DKD. Three major themes emerged from the interviews: (1) perceived potential benefits of proactive e-consults, (2) concerns about the proactive nature of e-consults, and (3) leveraging care teams to facilitate recommended DKD care.

### Theme 1: Potential benefits of proactive e-consults

#### Subtheme: Educational value

PCPs acknowledged the potential educational value of nephrology e-consults in facilitating delivery of guideline-recommended DKD care, particularly in the setting of rapidly evolving guidelines and limited early experience with new medications. The elements identified as potentially most high-yield to include in nephrology e-consults were specific medication recommendations (dosing, patient counseling, and how to monitor), diagnostic workup needed to establish CKD etiology, and guidance on when to refer a patient to nephrology. In addition, it was suggested that while concise, concrete recommendations were preferred, PCPs also would appreciate e-consults containing references to the practice guidelines behind recommendations, which could help them internalize new guideline changes and extend the knowledge in caring for other patients (Q1; Table 3). The ability to have two-way, “back-and-forth” interaction with nephrologists was also identified as a particularly valuable feature of e-consults for promoting learning:

As a PCP, I learn a lot from consultants. And I learn a lot more when there’s a back and forth with a specialist. And so like having an e-consult platform – incredibly helpful, right? Cause they don’t even need to see the patient necessarily. If I feel supported to start a medication, I have the parameters like what dose do I start, what’s my target dose, how do I get there, what monitoring do I need to do. What side effects do you commonly see? If I can get all that information from a specialist, I’m happy to do it. I don’t want to inconvenience a patient, and it’s a learning opportunity for me. (Q2; Table 3)

**Table 3:** Illustrative quotations for primary care provider perspectives on proactive e-consults

Site type	Quotation	Quotation No.
**Theme 1. Potential benefits of proactive e-consults**		
VA	I do think one of the cool things about e-consults is that they do serve…an educational and capacity building kind of function in addition to just providing that care for that one patient. Like if you can use it as a tool to teach the PCP like, these are 5 takeaways on KDIGO guidelines, because we know you don’t have time to read the whole thing…Just putting in, having it be clear, PCP, you only need to read these 2 lines, this is our ask of you. But here’s some other information should you care to know. These are the common side effects to look out for. This is how we typically do a titration, these are the labs we order and the frequency. I think it can subtly serve an educational function. (PCP17)	1
Safety net	As a PCP, I learn a lot from consultants. And I learn a lot more when there’s a back and forth with a specialist. And so like having an e-consult platform – incredibly helpful, right? Cause they don’t even need to see the patient necessarily. If I feel supported to start a medication, I have the parameters like what dose do I start, what’s my target dose, how do I get there, what monitoring do I need to do. What side effects do you commonly see? If I can get all that information from a specialist, I’m happy to do it. I don’t want to inconvenience a patient, and it’s a learning opportunity for me. (PCP1)	2
Academic	I think as primary care physicians, we try to do as much as we can and try to be very judicious with specialist referrals…we only want to make use of [specialists’] time if it’s really appropriate…I think sometimes we even push the boundaries of trying to handle everything by ourselves when in fact, it would actually benefit the patient to have a nephrologist review their case and give you recommendations without us having to do extra clicks and ask for it and type up the consult and do all that stuff unless it’s absolutely necessary. (PCP10)	3
VA	I think overall…lowering the activation energy from me having to say, ‘okay, do I want to bother my nephrology colleagues with this consult?’ If it were to automatically occur, I think that would decrease a lot of that activation energy. (PCP16)	4
Safety net	I’m thinking that there are some positive aspects of this, especially if I’m missing something for a patient. Of course there are things that haven’t been considered, so they can be helpful, especially if it’s gonna help optimize the care of the patient. (PCP5)	5
Safety net	I think most of us in primary care would really welcome the input. And you know, if only to be able to focus our efforts on trying to overcome the obstacles the patient faces in being adherent, instead of having all of our efforts being trying to figure out what’s the next best medicine or what should we be doing. So I think having that sort of clearly outlined would then allow us to focus our energy on trying to implement it instead of trying to figure it out. (PCP3)	6
**Theme 2. Concerns about proactive e-consults**		
VA	I think there’s probably the tail on the negative end with people who don’t like their information - like who value privacy and don’t want big brother to be looking through their chart and saying you need to be on this. So I think that’s a possibility, that’s a possible downside. (PCP16)	7
Safety net	I don’t think [patients] would mind it. I mean, it’s all part of our system of trying to improve things. I mean, in that if we had to sell it to the patients, that’s the way I would sell it, is that we’ve been doing this a lot, right? We’re all part of one big system that’ll work together to help you. (PCP4)	8
Safety net	I guess there might be people who feel like now that a specialist gave me recommendations, I have to abide by them and it’s another thing on my plate to do, and it’s in the chart. So it’s like, if I don’t follow these recommendations, then that’s like I’m providing substandard care. And it’s documented. (PCP8)	9
VA	Really, the only barrier [for proactive e-consults] would be if I had a sense of judgment, like why haven’t you started this empagliflozin on this patient yet? That would be like, well, because I’m too busy. So it would just be the friendly helpful suggestion. Framed that way, I can’t imagine that I would take that in any way as a barrier. (PCP16)	10
Safety net	I guess I could imagine a few sticklers being concerned that if there was a recommendation in the chart to do a certain thing, and then they didn’t do that certain thing, for whatever barrier, and then the patient ended up with a bad outcome, would they be legally at risk? (PCP3)	11
Safety net	It’s like, how do we create alerts that are actually impactful and that are not a nuisance, and don’t create alert fatigue. Cause just frankly, it’s just like an attention economy with anything like this. And you know everybody is having their attention pulled in a thousand different ways. (PCP1)	12
Academic	If I’m just getting this e-consult randomly, well, the next thing is…I need a visit with the patient, cause I’m either just going to forget it, or I need to get paid for enacting what might be some counseling and decisions. So either timing an e-consult with an upcoming visit, or… working with primary care to ensure anyone who gets an automated e-consult also gets booked for a visit with their PCP within a certain time period would, I think, do a lot to help. (PCP9)	13
**Theme 3. Leveraging care teams to facilitate recommended DKD care**		
Safety net	I think it’s still fine for the recipient to be the PCP…it’s more about just the operations of what happens after the PCP gets the message, and I think when people feel like you’re just pitting more on them…It’s a recipe for burnout. And so I think it may be more of like a training piece, and bringing these various stakeholders together, so that like, the director of ambulatory pharmacy has signed off on like…if you get these e-consults, you can feel free to forward it to your clinic pharmacist, and then they can reach out to the patient. Or maybe nursing does it, and they have a script for education that they provide…Like we have a lot of team resources in primary care, but I would say they’re not optimally utilized, and just a lot just falls on the PCP. And so the more we can unburden the PCP and say, hey, you’re gonna get this message, but we’ve gotten buy-in from the clinical pharmacy team that you can send this to them, and they can run with it. Just something like that can really go a long way. (PCP1)	14
Safety net	Some PCPs are very micromanaging, but I would say it’s pretty few and far between. I think people have moved away towards feeling like they need to make all of the decisions, and co-management with pharmacists is very common at this point. (PCP7)	15
VA	I would want to be notified, but I don’t necessarily think that the folks who’ve done due diligence around identifying proper medical optimization for these patients need my sign off on every single thing. I think that’s actually what makes our clinical pharmacy support so amazing is that anything that you can do to ease the cognitive load as the PCP, it usually makes things get done more efficiently and is more safe for patients in my opinion. (PCP15)	16
Safety Net	I don’t think I need to necessarily sign off on it. I do trust the pharmacists and the specialists that we work with…And then I think the FYI is mostly just so that I have an idea what’s happening. But I also understand that we get a lot of FYI’s in primary care…So, I could go either way on that. (PCP6)	17

*Abbreviations* DKD = diabetic kidney disease; KDIGO = Kidney Disease: Improving Global Outcomes; VA = Veterans Affairs

#### Subtheme: Improved access to specialists

PCPs described a degree of hesitance to initiating nephrology referrals, acknowledging high specialist workload and the perception that often, much of what would be done in nephrology clinic could be done in primary care (Q3; Table 3). Participants expressed that proactive e-consults could “lower the activation energy” for accessing nephrology expertise and could be a way to overcome PCPs’ hesitance to refer, particularly for patients with less severe kidney disease who may have unrecognized high-risk features (Q4; Table 3). In addition, participants noted e-consults could serve as a means to provide nephrology care for patients who face barriers to attending specialist appointments or prefer to see only their PCP.

#### Subtheme: reassurance of care plan

PCPs also saw value of proactive e-consults if they provided reassurance of the appropriateness of the existing care plan for each patient based on the most recent evidence and guidelines, and in particular that providers were not “missing anything” in the workup and treatment related to kidney disease (Q5; Table 3). In turn, this reassurance could allow PCPs to focus on working with patients to overcome obstacles to guideline-recommended care and to facilitate adherence, rather than considering whether they were overlooking any aspects to achieve optimal DKD outcomes:

I think most of us in primary care would really welcome the input. And you know, if only to be able to focus our efforts on trying to overcome the obstacles the patient faces in being adherent, instead of having all of our efforts being trying to figure out what’s the next best medicine or what should we be doing. So I think having that sort of clearly outlined would then allow us to focus our energy on trying to implement it instead of trying to figure it out. (Q6; Table 3)

### Theme 2: Concerns about proactive e-consults

#### Subtheme: Privacy concerns for patients

Participants expressed that the majority of their patients would likely receive proactive chart review by specialists positively as an opportunity to improve their health. However, there maybe be a small number of patients who would be less open to such a program due to privacy concerns, particularly regarding unsolicited chart review by providers they have not met without their explicit consent (Q7; Table 3). PCPs suggested that patients could be more accepting if the process were framed as a system-level effort to help ensure patients are getting recommended care:

I don’t think [patients] would mind it. I mean, it’s all part of our system of trying to improve things. I mean, in that if we had to sell it to the patients, that’s the way I would sell it, is that we’ve been doing this a lot, right? We’re all part of one big system that’ll work together to help you. (Q8; Table 3)

#### Subtheme: Appearance of substandard care

PCPs also raised the concern that e-consults could be viewed as documenting their delivery of substandard care in a way that is visible to others, potentially including patients:

I guess there might be people who feel like now that a specialist gave me recommendations, I have to abide by them and it’s another thing on my plate to do, and it’s in the chart. So it’s like, if I don’t follow these recommendations, then that’s like I’m providing substandard care. And it’s documented. (Q9; Table 3)

Several participants emphasized it was critical that proactive e-consult recommendations were written in a manner that would not feel punitive or judgmental when identifying patients not receiving guideline-recommended treatment, recognizing that there may be legitimate barriers to optimal care delivery in individual patients that PCPs are actively addressing (Q10; Table 3). Several participants also expressed concerns about potential medicolegal implications of proactive e-consults, particularly if specialist recommendations documented in an e-consult were not followed (Q11; Table 3).

#### Subtheme: Increased burden on PCP

PCPs at all sites raised concerns about the potential for proactive e-consults to increase the workload of already busy PCPs, who would then need to arrange a means of implementing the newly recommended care. They reported that clinicians were already familiar with receiving unsolicited input on patient care (e.g., automatically generated lists of patients needing vaccination or overdue for cancer screening), and that any new intervention would face competition for PCPs’ limited time in an “attention economy” (Q12; Table 3). To mitigate the time burden associated with proactive e-consults, multiple participants suggested that they be delivered shortly prior to patients’ upcoming appointments so that PCPs can see and implement the recommendations within the context of a patient visit (Q13; Table 3).

### Theme 3: Leveraging care teams to facilitate recommended DKD care

#### Subtheme: Clinic-based pharmacist or nurse could implement recommendations

PCPs in the VA and safety net practice settings identified a role for delegating implementation of guideline-recommended care from proactive e-consults to other clinic staff, such as a clinic nurse or ambulatory pharmacist, who are often already engaged with chronic disease medication management and panel management activities. This delegation would likely be more efficient and would mitigate the added time burden to PCPs:

I think it’s still fine for the recipient to be the PCP…it’s more about just the operations of what happens after the PCP gets the message, and I think when people feel like you’re just pitting more on them…It’s a recipe for burnout. And so I think it may be more of like a training piece, and bringing these various stakeholders together, so that like, the director of ambulatory pharmacy has signed off on like…if you get these e-consults, you can feel free to forward it to your clinic pharmacist, and then they can reach out to the patient. Or maybe nursing does it, and they have a script for education that they provide…Like we have a lot of team resources in primary care, but I would say they’re not optimally utilized, and just a lot just falls on the PCP. And so the more we can unburden the PCP and say, hey, you’re gonna get this message, but we’ve gotten buy-in from the clinical pharmacy team that you can send this to them, and they can run with it. Just something like that can really go a long way. (Q14; Table 3)

Meanwhile, PCPs at the academic health system tended to envision implementing e-consult recommendations personally, with minimal involvement from other clinic staff apart from scheduling an appointment to discuss DKD care. In addition, participants felt it was important for e-consult recommendations to be visible to all providers in the patient’s electronic health record, given the frequency of co-management and provider cross-coverage.

#### Subtheme: Desired level of involvement of PCP

PCPs varied in their preferences on their desired level of involvement with receiving and implementing proactive e-consults for DKD care. Participants acknowledged that most PCPs would want to be notified of potential medication changes but very few would feel the need to expressly sign off on implementation of e-consult recommendations, particularly in the context of co-management by clinical pharmacists (Q15 & 16; Table 3). Some participants even expressed ambivalence about the necessity of being notified as the PCP, citing the overwhelming volume of in-basket messages and the fact that any interim changes would eventually be reviewed by the PCP in subsequent visits:

I don’t think I need to necessarily sign off on it. I do trust the pharmacists and the specialists that we work with…And then I think the FYI is mostly just so that I have an idea what’s happening. But I also understand that we get a lot of FYI’s in primary care…So, I could go either way on that. (Q17; Table 3).

## Discussion

In our interviews with PCPs from three health systems, we found that PCPs were generally supportive of the concept of proactive nephrology e-consults for DKD management. PCPs identified common barriers to guideline-recommended DKD care delivery and noted potential benefits of having proactive guidance for overcoming some of these barriers and ensuring optimal DKD care. Meanwhile, PCPs identified key challenges for implementation of proactive e-consults and outlined potential mitigation strategies for successful integration into the primary care setting.

PCP support for proactive e-consults as a strategy to facilitate optimal DKD care delivery was largely in the context of its ability to guide the prescribing, counseling, and monitoring related to newer DKD medications, such as SGLT2 inhibitors, and its ability to assure that individual patients were getting appropriate kidney care. Furthermore, the recommendations offered in e-consults could serve an educational function and reinforce PCP knowledge and self-efficacy in caring for patients other than those who received e-consults [25]. Despite these potential benefits, however, PCPs acknowledged barriers to guideline-recommended DKD care that would not be addressed by proactive e-consults, in particular social risk factors. Such factors include patient low health literacy, challenges to attend clinic appointments regularly, and inability to afford medications. Even when PCPs are aware of the optimal DKD management and practice guidelines, other more immediate issues may take priority when patients are struggling with housing instability or substance use. Thus, while proactive e-consults have the potential to address some major barriers to optimal DKD care, they would not remedy all the barriers that PCPs identified in our study.

Comparing responses among PCPs practicing in three different health systems yielded some notable findings. Not surprisingly, the challenges to optimal care delivery were largely shared across health systems and have been documented previously [26, 27]. A notable contrast was found when PCPs were discussing how they envisioned how proactive e-consults for DKD management might be implemented in their practice. PCPs within the VA and safety net settings frequently referred to existing collaborations with clinic-based pharmacists and nurses as a logical resource for implementing e-consult recommendations. Since pharmacists and/or nurses were already so involved with medication management for patients with chronic diseases, leveraging their support for medication recommendations from proactive e-consults was felt to be a natural extension of the care they were already providing. Some PCPs even felt that e-consult recommendations could be sent directly to the nurse or pharmacist involved with a patient’s care, bypassing the PCP altogether. Meanwhile, PCPs in the academic health system tended to envision e-consults being followed up with an in-person visit in which the PCP could discuss directly with patients the e-consult recommendations. There, considerations such as timing proactive e-consults close to upcoming patient appointments was felt to be more crucial to ensuring the recommendations would be acted upon, otherwise they could risk becoming lost in volume of messages that PCPs receive daily.

Based on our results, several implications emerged for the design of a potential proactive e-consult intervention. First, it must be clear that the incorporation of proactive e-consults is intended to be supportive rather than critical, including non-judgmental language that could be perceived positively by clinicians and patients who have access to their electronic health record. PCPs should be informed and oriented to the purpose of proactive e-consults and the e-consult recommendations should avoid the implication that current care is substandard. The language for e-consult recommendations should be framed as a system-level improvement effort to optimize care delivery population-wide, rather than critiquing individual cases. Second, the implementation of a proactive e-consult program needs to be tailored to clinic workflows and resources available within health systems [28, 29]. Leveraging care teams such as clinic pharmacists or nurses could substantially improve the effectiveness of a proactive e-consult intervention compared with relying solely on PCPs to enact specialist recommendations. In addition, the recipient of proactive e-consults can be flexible and does not necessarily need to be the PCP. In determining these details of proactive e-consult design, it is crucial to involve and establish buy-in from all key clinic stakeholders and collaborate on a clear, agreed-upon protocol for handling e-consult recommendations.

Strengths of our study included exploration of PCP perspectives across diverse health systems, providing insights into the potential strategies for implementing proactive e-consults in practice settings with different workflows and resources. Limitations included the hypothetical nature of the interview questions, as responses may have differed among PCPs who had experience with proactive e-consult programs. Geographically, all three health systems were based in one city, thus limiting generalizability. Participants were predominantly female: while this may reflect demographic characteristics of local PCPs, it may also affect generalizability [21, 30]. We purposely allowed clinic directors to determine the most appropriate recruitment scheme, which varied across clinics, and as such we were unable to systematically collect characteristics of PCPs who were invited but did not participate. We focused the scope of our interviews to PCP perspectives across different health systems, but examining the perspectives of nephrologist and patient stakeholders on the acceptability of proactive e-consults will be a critical future direction. Additionally, we did not explore the potential financial considerations associated with the implementation of proactive e-consults. Traditional e-consults are sometimes a reimbursed clinical activity for which patients have a co-pay and require pre-authorization. While it is not clear how proactive e-consults would be funded across health systems, a traditional fee-for-service reimbursement mechanism could be inherently at odds with the nature of a proactive e-consult strategy.

## Conclusions

In summary, we found that PCPs saw potential benefits of proactive e-consults for DKD management, noting particular value from its ability to promote optimal treatment based on the most recent evidence and guidelines, reassure PCPs of the diagnosis and treatment plan, and serve an educational role to help PCPs stay up to date. PCPs also identified mitigation strategies for potential challenges in implementing proactive e-consults: recognizing the variability in workflows and resources between health systems, leveraging clinic support staff to enact e-consult recommendations, and framing e-consults as a system improvement effort rather than individual PCP critiques emerged as key considerations for successful implementation.

### Electronic supplementary material

Below is the link to the electronic supplementary material.

Supplementary Material 1

## Data Availability

The qualitative data for this study are not available as participants did not provide consent for complete transcripts to be made publicly available.

## Abbreviations

ACEi: Angiotensin converting enzyme inhibitor
ARB: Angiotensin II receptor blocker
CKD: Chronic kidney disease
CVD: Cardiovascular disease
DKD: Diabetic kidney disease
eGFR: Estimated glomerular filtration rate
KDIGO: Kidney Disease: Improving Global Outcomes
PCP: Primary care provider
SGLT2: Sodium-glucose cotransporter 2
VA: Veterans Affairs
