# Supplementary material for: Primary care clinician perspectives on automated nephrology e-consults for diabetic kidney disease: a pre-implementation qualitative study
Source: BMC Prim Care. 2024 Jun 4;25:197. doi: 10.1186/s12875-024-02454-w (PMC11149280; doi:10.1186/s12875-024-02454-w)
Supplement: Supplementary file 1 — Supplementary Material 1 [file 12875_2024_2454_MOESM1_ESM.docx]

**Supplementary Material: Primary care clinician perspectives on automated nephrology e-consults for diabetic kidney disease: a pre-implementation qualitative study**

Chi D. Chu MD MAS^1,2,3^, Daniel Dohan PhD^4^, Michelle M. Estrella MD MHS^1,3^, Michael G. Shlipak MD MPH^1,3^, Delphine S. Tuot MDCM MAS^1,2^

^1^ Department of Medicine, University of California, San Francisco, CA

^2^ Department of Medicine, Priscilla Chan and Mark Zuckerberg San Francisco General Hospital, San Francisco, CA

^3^ Kidney Health Research Collaborative, Department of Medicine, University of California, San Francisco, CA and San Francisco VA Health Care System, San Francisco, CA

^4^ Philip R. Lee Institute for Health Policy Studies, University of California, San Francisco, San Francisco, California, USA

**Supplementary Material Contents**

Item S1. Interview Guide for Primary Care Providers

**Item S1. Interview Guide for Primary Care Providers**

1. Tell me about your experience with taking care of patients with diabetes and chronic kidney disease (DKD).

a. Are you familiar with practice guidelines from any professional societies for DKD?

b. What do you find are challenges to providing guideline-concordant care for DKD?

c. Are there challenges related to specific medications? (e.g., ACEi/ARB or SGLT2 inhibitors)

d. How do you decide when to refer a patient with DKD to nephrology?

e. What is the referral process like?

A goal of this interview is to gather information to inform the development of a proactive e-consult intervention. This concept is inspired by the proactive e-consult mechanism at UCSF for inpatient glucose control, which involves an endocrinologist reviewing patients in the hospital flagged by the electronic health record for having severe hyperglycemia, and providing the primary teams with management recommendations. It is proactive in the sense that it does not require primary teams to initiate a consult. It also does not require primary teams to enact the specialist recommendations. We’re interested in seeing if a proactive e-consult concept could be used in the outpatient setting for improving management of DKD. A rationale for this approach, is that an algorithm could identify the highest risk patients not yet seen by nephrology and allow for two-way interaction with a specialist. This could promote more tailored recommendations for patients compared to a one-size-fits-all automated decision support pop-up or reminders about guideline-recommended medications.

2. What is your initial impression about the concept of proactive e-consults?

3. For patients with DKD, are there ways you see proactive e-consults might be helpful?

4. What do you think might be barriers or challenges to implementation of proactive e-consults?

a. Time – what do you foresee could be time-consuming if you are getting proactive e-consults?

b. Staffing issues

5. What kind of system or workflow changes might be needed to accommodate proactive consult implementation in your practice?

a. Are there ways that they could be made as helpful and unintrusive as possible?

6. How would you feel about receiving unsolicited recommendations for patients?

a. How do you think your peers might feel toward this concept?

b. How do you think your patients would feel about this concept?

c. Do you think such suggestions should be visible in the EHR for any provider and patient to see?

7. Overall, do you see value in the concept of proactive consults for DKD management?

a. What aspects would you find most valuable?

b. What would be necessary to maximize the chance of a successful intervention?

8. Before closing, are there any final thoughts or topics we didn’t cover that you would like to bring up?
